# Mechanistic investigation into the binding property of Yohimbe towards natural polymeric DNAs

**DOI:** 10.1038/s41598-023-40713-5

**Published:** 2023-09-19

**Authors:** Soching Luikham, Senchumbeni Yanthan, Jhimli Bhattacharyya

**Affiliations:** Department of Chemistry, National Institute of Technology Nagaland, Chumukedima, Nagaland 797103 India

**Keywords:** Biophysics, Chemical biology

## Abstract

DNA interactions with multivalent ligand(s) have increasingly become the subject of substantial research. For several small molecules with therapeutic potential, nucleic acids serve as their primary molecular target. Such interaction has been shown to affect transcription or replication, ultimately leading to apoptotic cell death. As a result, researchers are becoming increasingly interested in understanding how small molecules interact with DNA making it possible to develop new, DNA-specific drugs. The bioactive indole alkaloid, Yohimbe (Yohimbine; Yh) has been broadly studied in pharmacological properties while its binding mode to DNA has not been explicated so far. This study adopted molecular modelling and multi-spectroscopic methods to investigate the interaction between Yohimbine and herring testes (HT DNA) in physiological conditions. Minor hypochromic and bathochromic shifts of fluorescence intensity were observed, suggesting the binding of Yh to HT DNA. The Scatchard plot analyses using the McGhee-von Hipple method revealed non-cooperative binding and affinities in the range of 10^5^ M^−1^. The thermodynamic parameters suggested exothermic binding, which was favoured by negative enthalpy and positive entropy changes from temperature-dependent fluorescence experiments. Salt-dependent fluorescence suggested that the interaction between the ligand and DNA was governed by non-polyelectrolytic forces. The results of iodide quenching, urea denaturation assay, dye displacement, and in silico molecular docking, suggested groove binding of Yh to HT DNA. Thus, the groove binding mechanism of interaction was validated by both biophysical and computational techniques. The structural elucidation and energetic profiling of Yh's interaction with naturally occurring polymeric DNA can be useful to the development of DNA-targeted therapeutics.

## Introduction

**Figure 1:**
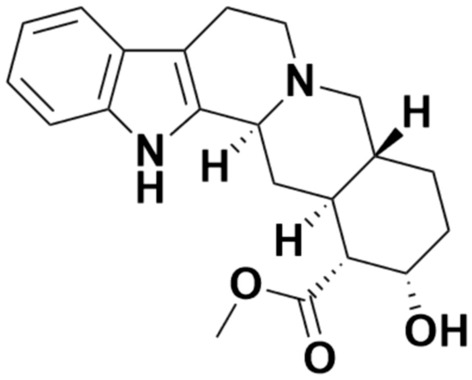
Chemical structures of Yohimbine (Yh).

Phytochemicals, over the years, have been receiving plenty of attention due to their pharmacological effects. One such phytochemical is yohimbine which has been mostly used as a stimulant and aphrodisiac for erectile dysfunction. Yohimbine (17α-hydroxyyohimban-16α-carboxylic acid methyl ester; Yh) (Fig. 1) is a plant alkaloid that is located in the bark of *Pausinystalia yohimbe*^1^ and has been used to treat various ailments. Symptoms that it has successfully treated include marijuana abuse, male erectile dysfunction, diabetes mellitus type II, orthostatic hypotension (low blood pressure when standing up), and depression^2–6^. It functions by blocking the pre-and postsynaptic α2-adrenergic receptors that are mainly responsible for treating erectile dysfunction. Additionally, yohimbine not only blocks the α-adreno receptor but is also able to enhance the sympathetic outflow from the CNS (central nervous system) as well as increase the release of catecholamine from the peripheral sympathetic nerve terminals. Yh belongs to the group of β-carboline molecules which have also marked impactful importance with their therapeutic importance^7,8^. These bioactive compounds are promising aspects in pharmacology and pharmacokinetics as they are abundantly present in nature and have lower toxicity as compared to other synthetically produced compounds. The interaction of these plant-derived compounds with nucleic acids is significant for developing various biomedical remedies^9–12^. Nucleic acids, specifically DNA (deoxyribonucleic acid) are important as it stores all the genetic information responsible for the therapeutic effect against cancer and various other diseases^13–17^. DNA is responsible for delivering genetic instruction to cells, assisting in cellular structure and function, and serving as a primary target of anticancer and antibiotics medications, etc.^18^. The structure of DNA has been well elaborated and thus makes it possible to study the effect of important chemical compounds on DNA in terms of pharmacology^19–26^. The fundamental goal while studying ligand–DNA interaction is the investigation of the type of binding. Generally, three types of interaction take place between ligands and DNA^27^: intercalative binding in which the ligands intercalate into the stacked DNA base pairs; groove binding occurs when ligands are bound on either of the two grooves of the double helix of DNA and electrostatic binding which takes place primarily between the negatively charged DNA phosphate backbone near the external DNA double helix and the cationic species. Notably, this interaction is non-specific. Groove or intercalative binding are two binding modes most likely to occur between the ligands which bind to the DNA non-covalently^27,28^. The interaction study of the molecule Yohimbine with HT-DNA (herring testes) was carried out to elaborate on the mechanism and thermodynamics involved in such types of binding studies. This study will help better understand the binding properties and analyze the varied structural and electronic aspects of the interaction. Analysis of DNA–ligand complexation is necessary to understand the molecular characteristics as well as the energy behind the complex formation^29–31^. A detailed understanding of this interaction study can help us in determining the potential therapeutic values that can be further utilized for the development of novel drugs. These investigations can also help us to characterize and define the forces that control, regulate, and stabilize this type of DNA–ligand interaction^32^. It also provides the overall details about the binding processes and the relationship between the structure and function of these ligands towards the biomacromolecules like DNA. Although in recent years, such biophysical studies have increased in the past decades however more scientific information is required to gain more insights into this type of study. The study of these nucleic acids–ligand systems using kinetic, structural, and thermodynamic methods helps in the overall build-up of databases which is instrumental for designing rational drug programs. For the first time, a comprehensive study has been done between Yh and HT-DNA using different spectroscopic techniques which include UV–Vis, fluorescence and molecular docking, etc. Thermodynamic parameters like binding constant (*K*), change in free energy (Δ°*G*), enthalpy (Δ°*H*), entropy (Δ°*S*), and stoichiometry (*n*) have also been taken into consideration.

## Materials and methods

### Materials

Yohimbine (Yh) and herring testes (HT) were purchased from Sigma-Aldrich Corporation. NaO_2_As(CH_3_)_2_·3H_2_O buffer containing 10 mM of sodium cacodylate was used for preparing samples and reactions, which have been done at a pH of 7.0. On a digital Systronics high-precision pH meter, pH readings, and corrections were conducted. Molar absorption coefficient (e) values of 13,100 M^−1^ cm^−1^ at 260 nm for HT were used to calculate the sample concentrations. After dissolving Yh in Millipore water, 1.0 × 10^–3^ Mol L^−1^ of Yh was made as the standard stock solution. Every essential reagent and chemical used in this investigation were of Sigma-Aldrich analytical grade. The experimental solutions were prepared with Millipore water.

### Methods

Using an Agilent Cary 100 range UV–Vis spectrophotometer at (298.15 ± 0.5) K, UV—Vis absorption experiments and binding stoichiometry (Job plot) analysis were carried out. On an Agilent Cary eclipse spectrofluorophotometer, fluorescence emission spectra, temperature-dependent, salt-dependent, potassium iodide (KI) quenching, urea denaturation, competitive dye displacement, and effect of metal ions were performed. Scatchard plotting studies were used to obtain the binding information from fluorimetric titrations. Molecular docking was carried out in AutoDock 1.5.6 using the Lamarckian Genetic Algorithm. The crystal structure of the DNA with the PDB-ID 1BNA for HT DNA was obtained from the Protein Data Bank. Every docked position was visualized with PyMOL (The PyMOL Molecular Graphics System, Version 2.3.4, Schrödinger, LLC) as well as the UCSF Chimera 1.15 molecular graphics tool. Details of the methods are discussed in Supplementary Information (S1).

## Results and discussion

### UV–Vis absorption determination

**Figure 2:**
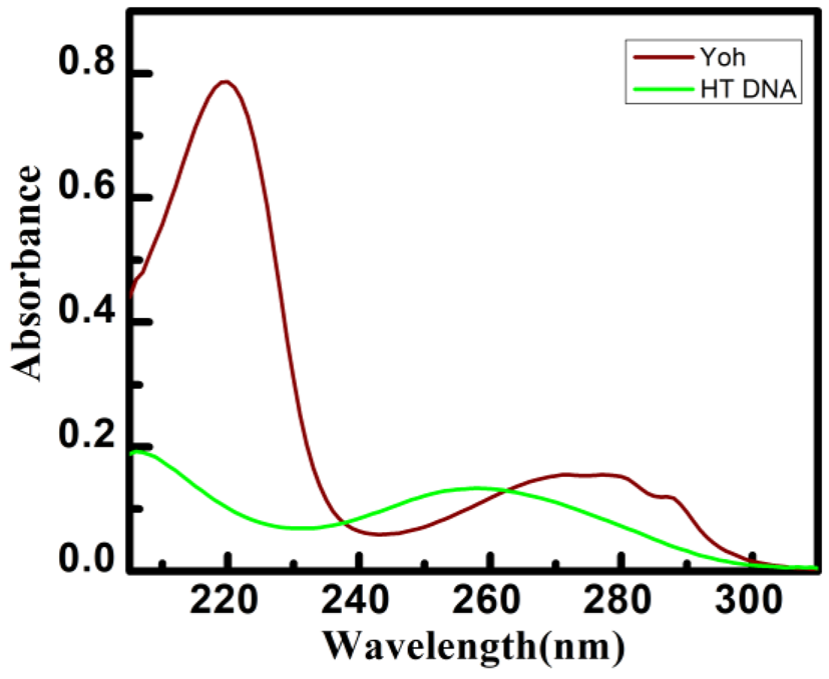
Representative absorption spectral changes of Yh (15 μM) treatment with HT DNA (15 μM). All experiments were performed in sodium cacodylate buffer (10 mM) of pH 7.0 at 298.15 K.

It is viable to monitor the interaction as Yh has a distinctive noticeable absorption spectrum in the 200–300 nm range with two significant peaks at 220 nm and 272 nm, respectively^33^. Similarly, Yh has also shown interactive properties with calf thymus DNA^34^. The electronic transition of the chromophore in both pyrimidines and purines was suggested as the basis of the maximum absorption of HT DNA at 260 nm^35^. Due to such spectrum overlap shown in Fig. 2, it was difficult to determine how Yh and HT DNA interacted. Upon interacting with HT DNA, it was found that Yh's UV absorbance at 220 nm decreased, but there was also a little hypochromic shift as well as a 1 nm bathochromic shift. This might be because of HT DNA base pair π orbital and the binding of ligand’s π* orbital together reduce the π–π* transition energy, causing an absorption red shift. The electron-filled coupling π*–orbital caused the hypochromic effect by reducing the rate of transfer^36^. Only the hypochromic effect with no or a very slight bathochromic shift can be observed when electrostatic and groove binding interactions occur^37^.

### Binding stoichiometry (Job's plot) analysis

**Figure 3:**
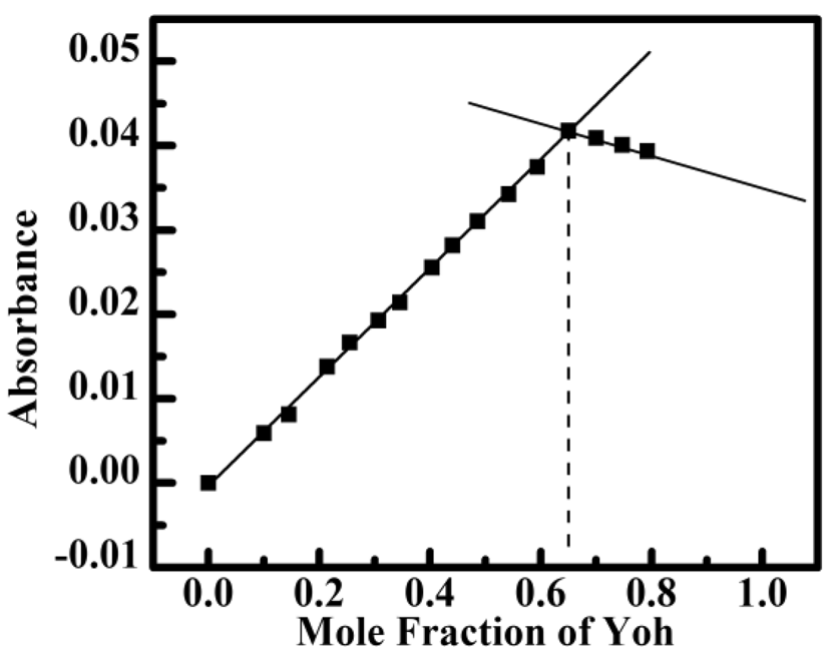
Job’s plot in absorbance plotted against mole fraction of added Yh to HT DNA at 298.15 K in sodium cacodylate buffer (10 mM) of pH 7.0.

In UV–Vis spectroscopy at a fixed temperature, a continual variation approach (Job's plot) was used to determine the Yh with HT DNA complex binding stoichiometry^38–40^. Job's plot analysis is a very useful technique for depicting the complex formed by the interaction of the two species. Variations in the Yh with HT DNA molar ratio were achieved while maintaining a fixed total molar concentration. The absorbance (260 nm) is plotted against the mole fraction of Yh in Fig. 3. The least-square fitted lines for HT DNA intersected at 0.65. The numbers correspond to the amount of HT DNA base pairs attached per molecule of Yh. This value indicates that, throughout the range of the investigated concentrations, the 2:1 binding stoichiometry for 2 Yh is spanned by 1 base pair of HT DNA. Similarly, Luikham et al. also obtained 2: 1 binding for Yh with calf thymus DNA^34^. The numbers of excluded sites derived from the McGhee–Von Hipple analysis of the fluorescence data and the values of stoichiometry are in close agreement.

### Fluorescence titration experiments

**Figure 4:**
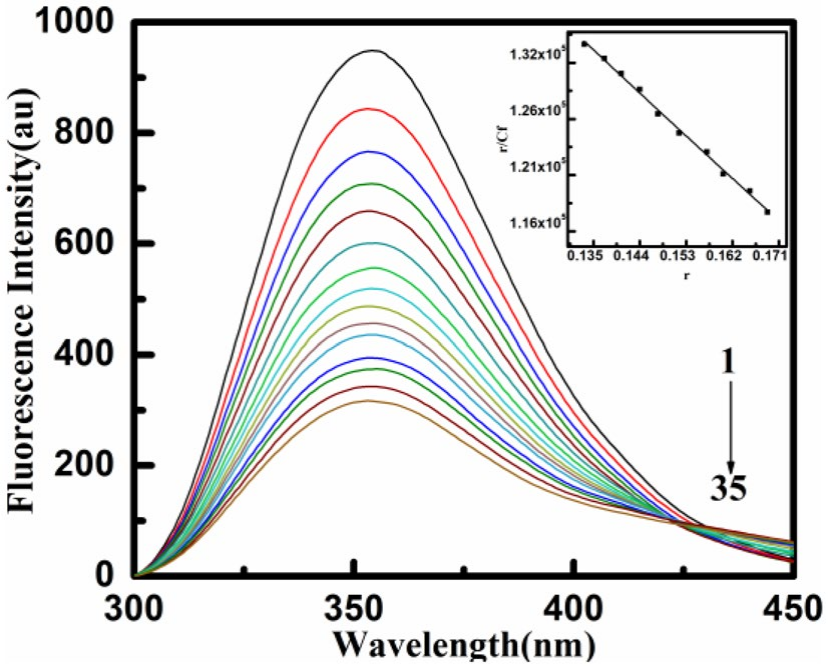
Representative spectral changes of fluorescence emission of Yh (15 μM) with an increase in the concentration of HT DNA (0.5 to 28 μM) at 298.15 K in sodium cacodylate buffer (10 mM) of pH 7.0. Inset: representative Scatchard plot of the binding of Yh with HT DNA.

An important method for determining how biomacromolecules and ligands interact is fluorescence spectroscopy^41,42^. Fluorescence experiments can be used to determine parameters like binding sites, dynamics, binding affinity, and conformational changes. Since DNA has relatively weak fluorescence, Yh was chosen as the fluorescence probe to analyze its interaction with DNA. The emission spectrum of Yh exhibits a strong intrinsic fluorescence and ranges from 300 to 440 nm, with maxima at 352 nm if excited at 250 nm. Fluorescence was quenched by interaction with HT DNA, which eventually caused the binding sites to be more saturated. Figure 4 depicts the complexation of Yh with HT DNA in comparison to other fluorescence characteristics. The fluorescence quenching was approximately about 60%, which indicates that the ligand has a strong affinity for these natural polymeric DNA structures. Following the application of McGhee-von Hippel analysis, this data were utilized to create linear graphs of the Scatchard binding isotherm (inset Fig. 4), which revealed non-cooperative binding^43^. The binding constants determined were (*K*)^43–47^ of 2.00 × 10^5^ M^−1^ for HT DNA at 10 mM, T = 298.15 K. Therefore, the fluorescence data likewise substantially indicate that Yh binds moderately to HT DNA. The “*n*” value, which represents the relevant number of binding sites, was 1.8.

### Temperature dependent fluorescence spectral study

Through fluorescence titration with a constant drug concentration with increased DNA concentration at 288.15 to 308.15 K, the thermodynamic binding of Yh with HT DNA was observed. It also presents an important understanding of the molecular forces that accelerate the formation of complex^48^. The significance is that it can be used to estimate the stoichiometry, ligand binding constant, and complete thermodynamic profile of the interaction. The experimental binding constant of Yh with HT DNA (Figs. S1 & S2) at temperatures between 288.15 and 308.15 K is represented in Table 1. Results revealed that with an increase in temperature from 288.15 to 308.15 K, the binding affinity of such interaction lowered, resulting in a moderately weak binding of Yh with HT DNA. A fall from 2.20 × 10^5^ M^−1^ to 1.59 × 10^5^ M^−1^ at 308.15 K occurred (Table 1), which determines the ligand–DNA complexation was disrupted due to the increase in temperature. The '*n*' value (binding site), was approximately 1.8 for all of the temperatures condition. However, there was some significance in the thermodynamic analysis. Throughout all temperatures, the Gibbs free energy is negative indicating that the reaction is spontaneous.

**Table 1:** *K* (binding constant) and *n* (binding sites) for Yh with HT DNA interactions at different temperatures.

Temp (K)	Yh–HT	
	*K* × 10^5^ (M^−1^)	*n*
288.15	2.20 ± 0.01	1.8 ± 0.06
293.15	2.10 ± 0.04	1.8 ± 0.08
298.15	2.00 ± 0.03	1.8 ± 0.09
303.15	1.66 ± 0.02	2.1 ± 0.36
308.15	1.59 ± 0.01	1.7 ± 0.07

All experiments were carried out in sodium cacodylate buffer (10 mM) of pH 7.0.

**Figure 5:**
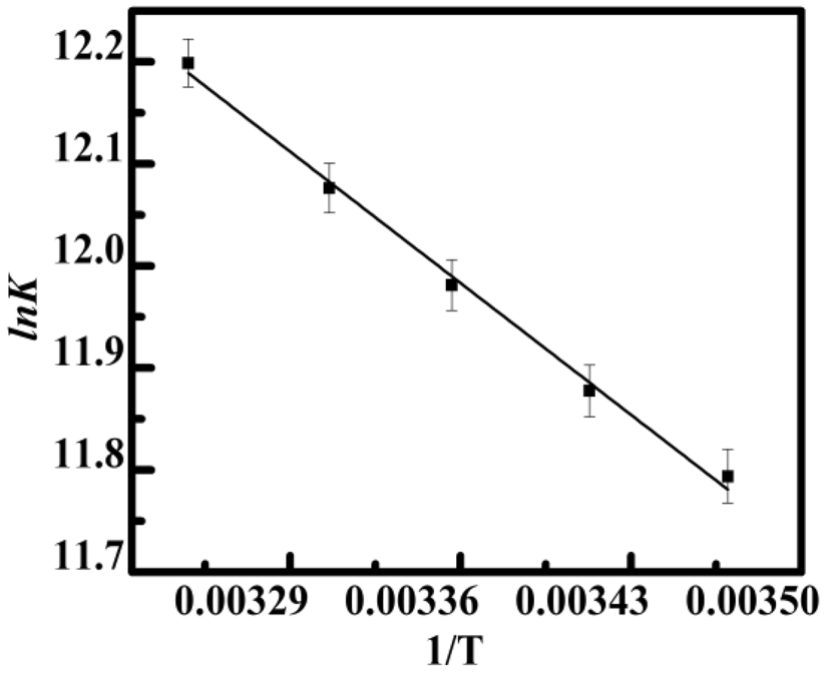
Van’t Hoff Plot of *lnK* versus *1/T* of Yh with HT DNA respectively. All experiments were carried out in sodium cacodylate buffer (10 mM) of pH 7.0.

Van der Waals forces, hydrogen bonds, hydrophobic forces, and electrostatic interactions are the primary force that links small molecules and biomolecules^37^. You may assess the underlying forces for a ligand–DNA interaction to develop by observing the variations throughout the enthalpy (Δ*H*°), entropy (Δ*S*°), and free energy (Δ*G*°) values for ligand–DNA binding. Thus, the total Gibbs free binding energy (G) from the Gibbs–Helmholtz model and the classical van't Hoff formula was used to establish the thermodynamic properties for Yh with HT DNA system at 288.15 to 308.15 K:

Δ*H*°, Δ*S*°, and Δ*G*° can be described from the following Eqs. (1) and (2).

1 $$lnK = \, {-}\Delta H^\circ /RT + \Delta S^\circ /R$$

2 $$\Delta G^\circ = \, \Delta H^\circ {-}T\Delta S^\circ_{\hbox{-}} = {-}RT \, lnK$$

where R (8.314 J mol^−1^ K^−1^) is the universal gas constant and *K* is the binding strength at the suitable temperature. Equation (2) can be used to determine the free energy change. Δ*H*° and Δ*S*° values were determined by calculating the slope (–Δ*H*°*/R*) and the intercept (Δ*S*°*/R*) of the plot using Eq. (1) based on *lnK* versus *1/T* (Fig. 5). Thus variables of Δ*G*°, Δ*H*°, and Δ*S*° were calculated using Eqs. (1) and (2), and the findings are presented in Table 2. In accordance with the report, the primary force includes electrostatic when Δ*H*° < 0 and Δ*S*° > 0, the essential factor is hydrogen bonding and Van der Waals when Δ*H*° < 0 and Δ*S*° < 0, and the major force is hydrophobic association when Δ*H*° > 0 and Δ*S*° > 0^49^. According to Table 2, which shows the thermodynamic characterization of such interaction, the complexation favors negative enthalpy, with Δ*H*° = – 13.01 kJ mol^−1^ (Yh to HT DNA), and a significant positive entropy contribution, with Δ*S*° = 17.28 kJ mol^−1^. The results imply that the chemical bonding is driven by both hydrogen bonds, and hydrophobic interactions, predominantly electrostatic forces with an exothermic binding process driven by entropy, which is a hallmark of ligand–DNA complexation^47,50^. The binding process was spontaneous as a result of the shift in negative free energy (Δ*G*°) throughout all temperatures (Δ*G*° < 0).

**Table 2:** Temperature-dependent thermodynamics parameters for binding of Yh to HT DNA.

Temp (K)	Yh–HT		
	Δ*G*° kJ mol^−1^	Δ*H*° kJ mol^−1^	TΔ*S*° kJ mol^−1^
288.15	− 30.29 ± 0.06	− 13.01 ± 0.36	17.28 ± 0.04
293.15	− 30.59 ± 0.01		17.58 ± 0.09
298.15	− 30.89 ± 0.02		17.88 ± 0.03
303.15	− 31.19 ± 0.08		18.18 ± 0.02
308.15	− 31.49 ± 0.03		18.48 ± 0.07

All experiments were carried out in sodium cacodylate buffer (10 mM) of pH 7.0.

### Salt dependent fluorescence spectral study

To comprehend the origin of such molecular forces governing the binding process, salt-dependent fluorescence studies were carried out^51^. Together with Van't Hoff analysis carried out in fluorescence at five varying concentrations of salt^41^. Around the double helix of DNA, cations remain available forming counter ions, and ligands attempt to release these cations to neutralize the phosphate backbone. Both mechanisms are thermodynamically related. Results outcomes from salt-dependent fluorometric experiments are shown in Table 3. When [Na^+^] increased, the values of *K* (equilibrium constant) decreased, showing complexation was destabilized at a greater salt concentration (Figs. S3 and S4). As a result, the quantity of salt in the environment had a significant effect on how strong the interaction is. Their values of ‘*n*’, meanwhile, maintained relatively consistent and indicated a 2:1 complex formation between the possible binding molecules under all salt concentrations.

**Table 3:** *K* (binding constant) and *n* (binding sites) for Yh to HT DNA interactions at different salt concentrations.

[Na^+^] (mM)	Yh–HT	
	*K* × 10^5^ (M^−1^)	*n*
10	2.00 ± 0.25	1.8 ± 0.03
20	1.97 ± 0.04	1.6 ± 0.02
30	1.29 ± 0.12	1.8 ± 0.02
50	1.12 ± 0.05	1.1 ± 0.04
100	1.10 ± 0.17	1.4 ± 0.24

Temperature = 298.15 K.

Most ligand–DNA binding interactions depend on electrostatic interactions. Thus, according to Chaires and his coworkers' study, the binding free energy was divided between polyelectrolytic and non-polyelectrolytic portions. Researchers used a fluorescence experiment as well as a Van't Hoff analysis to compare the effects of the salt concentration from the range of [Na^+^] 10 mM to 100 mM. The binding capacity of such contact decreased as the [Na^+^] level increased. A greater fall from 2.00 × 10^5^ M^−1^ at 10 mM to 1.10 × 10^5^ M^−1^ at 100 mM occurred (Table 3), A rise in [Na^+^] quantity reduces total electrostatic interactions between the phosphate groups with negative charges of consecutive nucleotides of DNA, which may hamper the interaction of such a ligand and cause a reduction in binding affinity levels as seen in Table 3. Manning's counter ions model-based polyelectrolytic theories just describe the process and provide a framework for interpreting the subsequent findings^52^. The best fit linear slope of such plot of *lnK* versus *ln[Na*^+^*]* is connected to the counter ion liberated based on the polyelectrolytic theory^53^ by the given relation

3 $$SK = ln \, (K)/ln([Na^{ + } ]) = {-}Z\Psi$$

**Figure 6:**
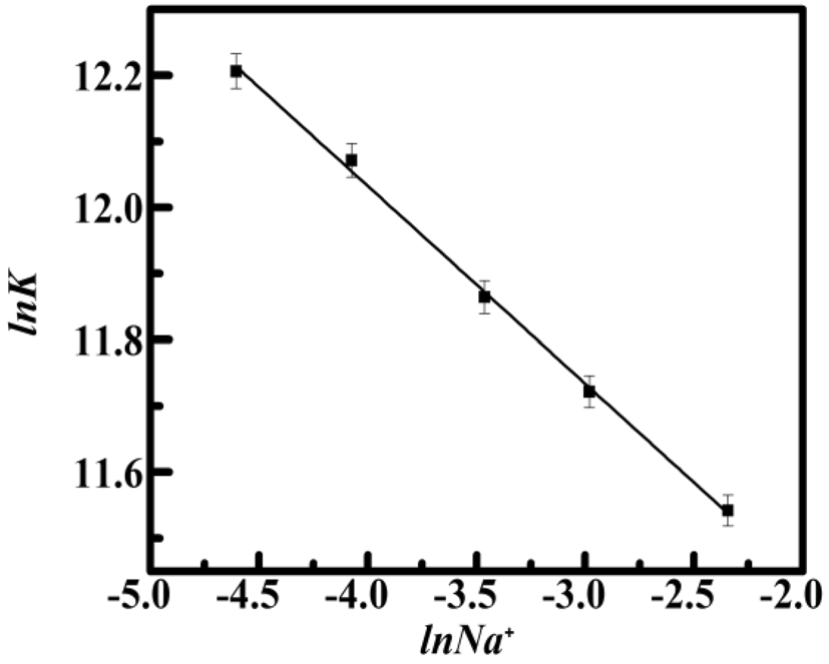
Van’t Hoff Plot of *lnK* versus *ln[Na*^+^*]* of Yh with HT DNA respectively. All experiments were carried out in different salt concentrations of sodium cacodylate buffer of pH 7.0, Temperature = 298.15 K.

Here *Z* is the actual value of the ligand-bound per phosphate binding, *Ψ* is the proportion of [Na^+^] bound per phosphate group, and *SK* is the number of counter ions linked to the drug complexation. Figure 6 slope of the *lnK* versus *ln[Na*^+^*]* graph indicates results of 0.31. Significant evidence for this is provided by dividing the observed binding Gibbs free energy into components from polyelectrolytic (Δ*G°*_*pe*_) and non-polyelectrolytic (Δ*G°*_*t*_) processes. This relation shown below can be utilized to quantify its polyeletrolytic component towards the expected change in free Gibbs energy

4 $$\Delta G^\circ_{pe} = {-}Z\Psi RT \, ln\left( {\left[ {Na^{ + } } \right]} \right)$$

**Figure 7:**
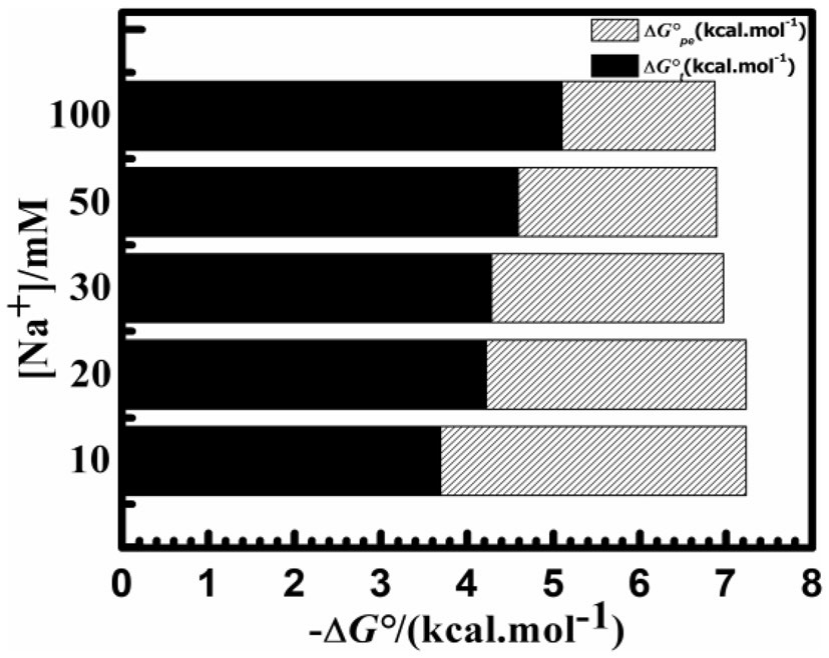
Partitioned polyelectrolytic (Δ*G°*_*pe*_) (shaded) and non-polyelectrolytic (Δ*G°*_*t*_) (black) presented to the Gibbs free energy change at (10, 20, 30, 50, 100) mM [Na^+^] concentrations HT DNA respectively. Temperature = 298.15 K. All experiments were carried out in different salt concentrations of sodium cacodylate buffer of pH 7.0.

The non-polyelectrolytic Δ*G°*_*t*_ component is defined by the difference between Δ*G°* and Δ*G°*_*pe*_ at specific [Na^+^] concentrations. The Van't Hoff plot's slope is represented here by ZΨ (see Fig. 6). The proportion of Δ*G°*_*pe*_ towards the overall changes in Gibbs free energy at 10 mM [Na^+^] was determined to be − 3.54 kcal mol^−1^ and about 25%. Estimated values of Δ*G°*_*pe*_ were calculated to be − 1.77 kcal mol^−1^ for salt content at 100 mM [Na^+^], which is nearly 15% of the total change in Gibbs free energy. Figure 7 and Table 4 show a depiction of the divided Gibbs-free energy change. It is evident that each time, whenever the [Na^+^] concentration rises, the Δ*G°*_*t*_ component stayed constant while the Δ*G°*_*pe*_ component is reduced. This complexation by Yh binding to HT DNA is stabilized mainly by non-polyelectrolytic (Δ*G°*_*t*_). These outcomes presented therein serve as yet another affirmation of the part non-polyelectrolytic factors play throughout the complex formation. The non-polyelectrolytic component to the binding of Yh to HT DNA is considerably greater than that of the polyelectrolytic (Δ*G°*_*pe*_) component.

**Table 4:** Salt-dependent thermodynamics parameters for binding of Yh–HT.

[Na^+^] (mM)	Yh–HT			
	Δ*G*° kcal mol^−1^	Δ*G*_*t*_° kcal mol^−1^	Δ*G*_*pe*_° kcal mol^−1^	ZΨ
10	− 7.23 ± 0.08	− 3.69 ± 0.21	− 3.54 ± 0.17	0.31 ± 0.01
20	− 7.22 ± 0.09	− 4.22 ± 0.25	− 3.01 ± 0.16	
30	− 6.97 ± 0.36	− 4.28 ± 0.04	− 2.69 ± 0.03	
50	− 6.89 ± 0.07	− 4.59 ± 0.12	− 2.3 ± 0.01	
100	− 6.87 ± 0.03	− 5.1 ± 0.05	− 1.77 ± 0.01	

All experiments were carried out at a different salt concentration of pH 7.0, Temperature = 298.15 K.

### Potassium iodide (KI) quenching experiments

**Figure 8:**
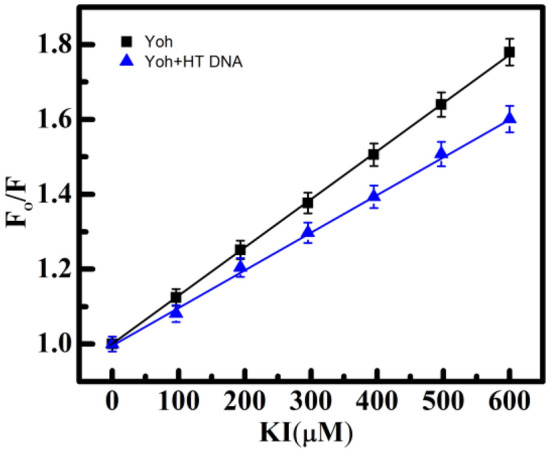
The Stern–Volmer plot of fluorescence emission of Yh titrated with KI (10–600 μM) in the presence and absence of HT DNA (50 μM). Temperature = 298.15 K. All experiments were carried out in sodium cacodylate buffer (10 mM) of pH 7.0.

This quenching compound called potassium iodide (KI) was utilized to confirm the way that Yh interacts with HT DNA. Small molecules which are intercalated inside DNA's helical structure are shielded from being quenched because of an ionic quencher since the negatively charged DNA phosphate backbone is likely to oppose a strongly negatively charged quencher. Additionally, ionic quenchers easily quench those groove-binding molecules that remained in proximity to the surroundings^54^. As a result, after titrating using KI, the observed range in K_sv_ values is greater for intercalating chemicals than for molecules that bind to grooves^55^. In 3 sets of tests, both emission spectra of Yh alone and Yh to HT DNA complexes after titration with KI were determined. Through the use of the following formula, the K_sv_ values were derived from the Stern–Volmer plot (Fig. 8 and Table 5):

5 $${\text{F}}_{{\text{o}}} /{\text{F}} = {1} + {\text{K}}_{{{\text{sv}}}} \left[ {{\text{KI}}} \right]$$

**Table 5:** The binding constants of Yh with HT DNA complex with the addition of common metal ions.

	HT DNA K_sv_ (× 10^2^ M^−1^)
Yh	1.12 ± 0.12
Yh–Ca^2+^	0.83 ± 0.32
Yh–Mg^2+^	0.85 ± 0.05
Yh–K^+^	0.94 ± 0.24
Yh–Zn^2+^	0.65 ± 0.76

All experiment was carried out in sodium cacodylate buffer (10 mM) of pH 7.0.

Where F_o_ and F indicate the emission intensities in the presence and absence of the quencher, respectively (KI). The K_sv_ values of Yh alone and Yh to HT DNA were found to be 1.3 ± 0.21 × 10^3^ M^−1^, and 1.02 ± 0.34 × 10^3^ M^−1^ respectively. The emission of Yh after and before the interaction with HT DNA was substantially similar in the results, showing little variation in the iodide quenching effect. Thus, Yh's groove-binding mechanism with HT DNA is implemented^46,55,56^.

### Urea induced assay

This assay is often carried out to confirm how small molecules interact with natural polymeric DNA. The dsDNA helix is destabilized by denaturants like urea, which also causes the release of small-intercalated ligands from DNA and alters the fluorescence emission intensity of small molecules^57^. With the addition of urea, essentially no change is made to the photoluminescence intensity of groove-binding ligands.

**Figure 9:**
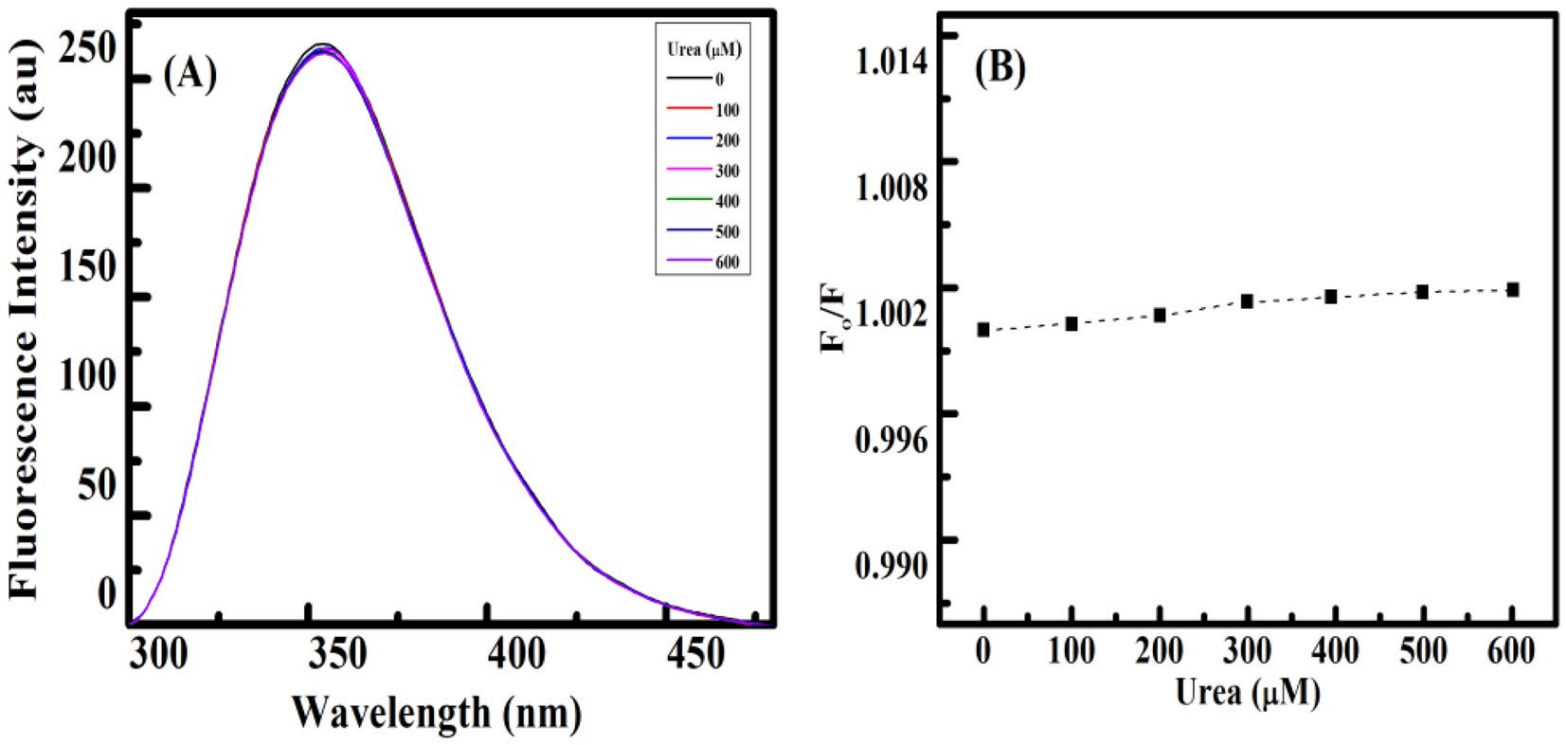
Fluorescence intensity spectra of Yh with (**A**) HT DNA titrated with urea (0–600 μM), (**B**). The plot of F_o_/F versus concentration of urea HT DNA. Temperature = 298.15 K. All experiments were carried out in sodium cacodylate buffer (10 mM) of pH 7.0.

Following treatment using urea, this was noted that the intensity of the fluorescence remained constant (Fig. 9B), indicating the groove binding mechanism instead of the intercalation mechanism^58^. Figure 9A depicts the relationship between the emission intensities of the Yh to HT DNA complex in the addition and exclusion of urea (F_o_/F) as a result of urea concentration. A similar result has been obtained of Yh when interacting with calf thymus DNA^34^. Therefore, our result supports the groove binding mode.

### Competitive drug displacement assay

These fluorescent dye tests were conducted to confirm how a small molecule binds to natural polymeric DNA. For instance, ethidium bromide attaches to DNA through an intercalative association while rhodamine-B binds via a groove binding mode^59–61^. The most common groove binding markers and intercalative binding markers are rhodamine-B and ethidium bromide, respectively. Rhodamine-B and ethidium bromide were utilized as binding indicators in competitive binding studies to even further confirm the binding mechanism of Yh with HT DNA. The shift in the emission spectra of such dye–DNA complex formation is monitored when the dye–DNA complex would be enabled to bind to Yh, its binding mechanism needs to be determined. As Yh competes with the dyes bound to DNA, it is therefore expected that it would interact with the DNA double helix in a manner that is similar to that of the dye it replaces^62^.

**Figure 10:**
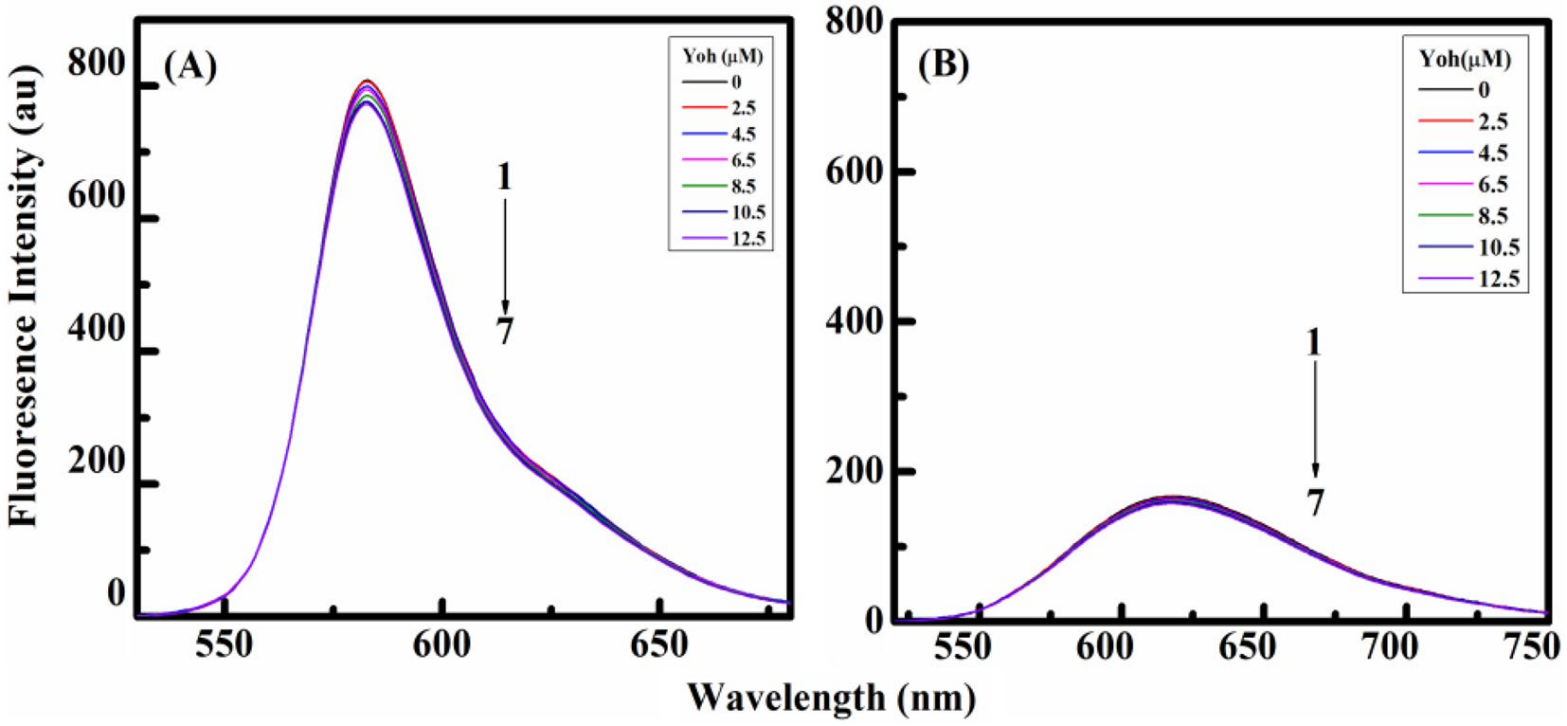
Fluorescence emission spectra obtained upon titration with Yh (0‒12.5 μM) on the complex between HT-DNA and (**A**) Rhodamine-B and (**B**) Ethidium bromide. Temperature = 298.15 K. All experiments were carried out in sodium cacodylate buffer (10 mM) of pH 7.0.

Fluorescence spectra were obtained while the dyes to HT DNA combination were determined by titration with increasing amounts of Yh in various sets of assays. When HT DNA interacts with rhodamine-B and was titrated with Yh, fluorescence appeared to be quenched (Fig. 10A), but ethidium bromide had no such impact (Fig. 10B). K_sv_ values from the Stern–Volmer plot were used to determine the shifts in emission intensity of such dyes after they were attached to HT DNA by Yh. To further confirm the groove binding mode the experiment was also performed with acridine orange which is an intercalator and further no shifts or significant change was observed (see Supplementary Fig. S5). Rhodamine-B has substantially higher K_sv_ values than ethidium bromide (Fig. 10). Our data corroborate how Yh attaches to HT DNA through a groove binding mode based on the aforementioned results obtained^63,64^. This is in agreement with the above mentioned experimental outcomes.

### Effect of metal ions

Vitamins and minerals found in food items may increase or decrease the drug's pharmacokinetic profile (absorption, dispersion, digestion, or elimination) effects. Our human body requires metal ions to stay in good health. They take part in a variety of biological processes as co-factors^65^. In the current study, the binding influence of metal ions (such as Mg^2+^, Zn^2+^, Ca^2+^, and K^+^) on Yh with HT DNA was examined by keeping the concentrations of Yh (5 μM) and metal ions (2.5 μM) and varying the amount of HT DNA (0–15 μM). In Table 5, the determined bond quenching values (K_sv_) for the absence and presence of metal ions are presented. It is noticeable that when ions were added, the K_sv_ values were altered. The interaction with both Yh and the metal ion could be responsible for this alteration.

Complexation of Yh with a metal ion could be the cause for the increase in K_sv_. Increased K_sv_ values could increase Yh's effect and retention time. Whereas, the interaction of HT DNA with metal ions, results in complextion, leading to decreased K_sv_ values. Such complexation is anticipated to have an influence on the structure of the DNA and the dynamics of Yh interaction. Therefore, the elimination rate of Yh may slightly increase (decrease in K_sv_), since the metal ions inhibit ligand binding or promote ligand dissociation^65^. The decrease in K_sv_ was in the order of, Zn^2+^ > Ca^2+^ > Mg^2+^ > K^+^.

### Molecular modeling results

**Figure 11:**
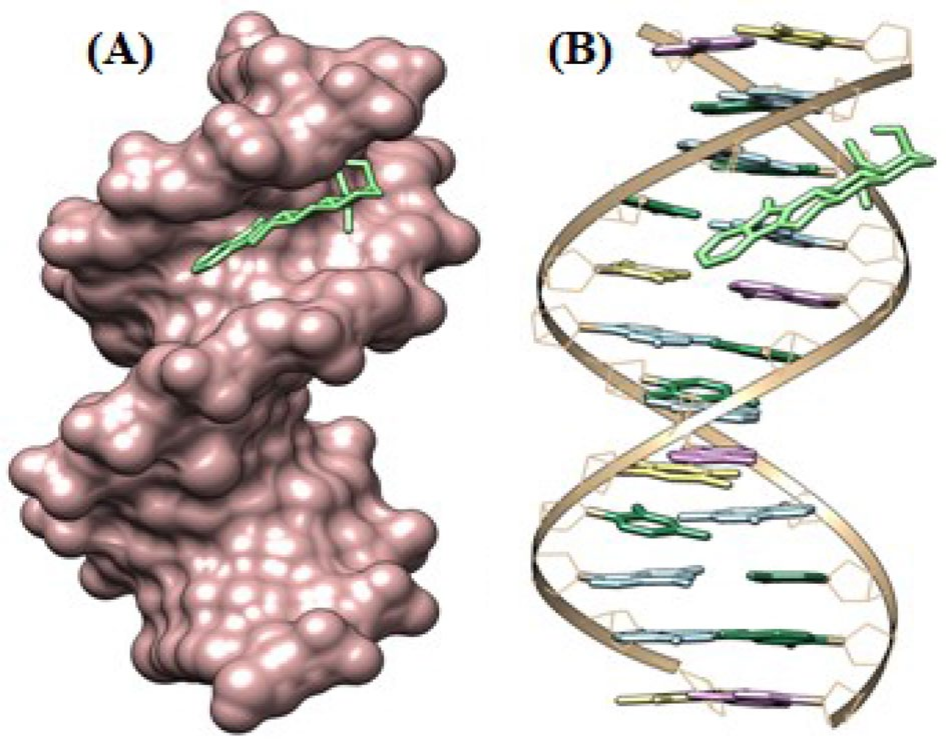
(**A**) Docked pose of Yh with HT DNA (PDB ID: 423D), (**B**) Hydrophobic surface of HT DNA (423D).

Information regarding the drug's interaction with DNA upon binding is revealed through molecular modelling study. To understand the binding interactions, docking of the lowest energy conformer of Yh was performed with HT DNA (PDB-ID; 423D) using the AutoDock 1.5.6 program^45,66^. In each instance, docking was employed to highlight the Yh binding interaction with the HT DNA and to comprehend the underlying forces that are involved. The Yh compound was confirmed to exhibit a clear interaction with the naturally occurring polymeric HT DNA, as shown in Fig. 11A and B. During the interaction of Yh with HT DNA, electrostatic energy is much lower than that of the total of hydrogen bonding, Van der Waal's energy, and desolvation-free energy.

**Figure 12:**
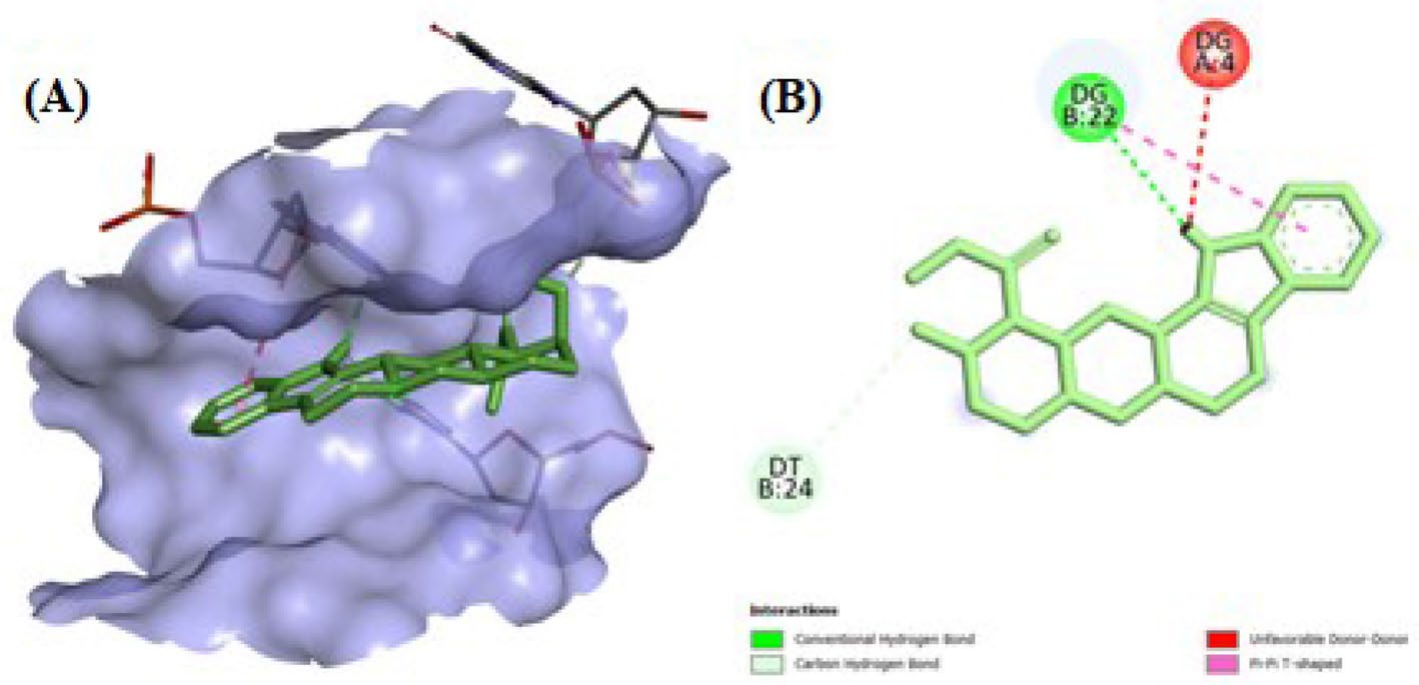
(**A**) Hydrophobic forces covering the surface of HT DNA (423D) with Yh, (**B**) 2D diagram indicating the DNA base pairs of HT DNA (423D) with Yh and interacting forces in the complex formation.

In every case, hydrogen bonding (H-bond) was present. With a separation of 1.77 Å, the indole part of Yh formed an H-bond with the C_3_-NH of the dihydropurin group of guanine (Fig. 12A,B). Researchers have also performed molecular docking of Yh with hemoglobin protein^33^. It is estimated that Yh can attach to HT DNA with a binding energy of − 11.2 kcal mol^−1^. The experimental findings are corroborated by the expected binding energy using molecular modelling. The inhibition constant was 6.81 μM. Thus, the mode of binding was via groove binding which also complements the experimental data.

## Conclusion

In this study, a series of techniques, including multifaceted spectroscopy and molecular modeling methods, were utilized to investigate the binding affinity, binding mode, energitics, and structural aspects of a versatile indole alkaloid drug Yohimbine (Yh) with natural polymeric DNA (herring testes DNA). The complex formation, by Yh with the HT DNA, was substantiated by the analyses of UV absorption and steady-state fluorescence spectroscopy. Fluorescence spectrophotometric results and calculations on the binding constants revealed that Yh binds to HT DNA effectively with 2:1 complex formation (stoichiometry). According to the structural elucidation data, electrostatic forces were the key factors by which Yh attached to HT DNA in the groove site; with a binding constant in the order of 10^5^ M^−1^. Furthermore, one (01) hydrogen bond was generated at a length of 1.77 Å between the C_3_-NH of the dihydropurin group of guanine and the indole part of Yh, as confirmed by molecular modeling analyses. Job plot analyses confirmed the 2:1 binding for Yh on DNA which also supports the fluorescence data. Potassium iodide quenching, urea-induced denaturation assay as well as dye displacement studies unequivocally established that Yh binds in the DNA groove region, which was also supplemented by computational analyses. The thermodynamic association between Yh and HT DNA was demonstrated using various temperature-based fluorescence and molecular modeling approaches. This interaction was exothermic, and the binding was facilitated by both negative enthalpy and positive entropy changes. Studies utilizing salt as a factor revealed that hydrophobic molecules dominated the binding with non-polyelectrolytic forces. The groove-binding mechanism of Yh with natural polymeric DNA was computationally validated, and also the findings of the experiment(s) were therefore supported by the molecular docking analyses. The findings provided above may give pharmaceutical scientists additional knowledge as they continue to work on creating novel DNA-based therapeutic medicines. It was determined that the heat created by increasing temperatures unwinds DNA strands, allowing Yh to attach to HT DNA. The above findings reveal specific information on the complex formation which is important for logical personalized medicine, such as the binding constant, binding site, binding mode, and types of interacting forces. Thus, Yh can be a potential alkaloid-based drug with selective properties and greater efficacy in the future.

### Supplementary Information

Supplementary Figures.

## Data Availability

All data are available in the main text or the supplementary materials.
