# Supplementary material for: Mechanistic investigation into the binding property of Yohimbe towards natural polymeric DNAs
Source: Sci Rep. 2023 Sep 19;13:15487. doi: 10.1038/s41598-023-40713-5 (PMC10509242; doi:10.1038/s41598-023-40713-5)
Supplement: Supplementary file 1 — Supplementary Figures. [file 41598_2023_40713_MOESM1_ESM.docx]

**Mechanistic investigation into the binding property of Yohimbe towards natural polymeric DNAs**

Soching Luikham, Senchumbeni Yanthan, and Jhimli Bhattacharyya^*^

Department of Chemistry, National Institute of Technology Nagaland, Chumukedima, Nagaland, 797103, India

Corresponding Email ID: jhimli@nitnagaland.ac.in

**Electronic Supplementary Information (ESI)**

**Methods**

*UV-Vis Absorption Spectroscopy*

An Agilent, Cary 100 series UV-Vis spectrophotometer in standard quartz cuvettes of 3.5 ml with a 10 mm optical path length was used to analyze the absorption spectral studies at (298.15 ± 0.5) K. For the drug–DNA study, before recording the absorbance values, the solution was properly mixed and allowed to change state after each fraction of the drug was added to the DNA solution. At a minimum, three continuous measurements were taken for each sample and averaged out.

*Binding stoichiometry (Job Plot) analysis*

The continuous variation technique was employed to confirm the binding stoichiometry ratio in each case using UV-Vis spectroscopy. All the measurements were carried out at a fixed temperature of 298.15 K. The absorbance signal was recorded for solutions where the concentrations of both DNA and the alkaloid were varied while the sum of their concentrations was kept constant. As a function of the input mol fraction of each alkaloid, the difference in each alkaloid's absorbance in the presence and absence of DNA was plotted. The mol fraction of the complex's bound alkaloid, which is represented by the breakpoint in the resulting plot. The binding stoichiometry was obtained in terms of DNA-alkaloid [(1-χ_alkaloid_)/ χ_alkaloid_] where χ_alkaloid_ denotes the mole fraction of the alkaloid. The results reported are average of at least three experiments.

*Fluorescence Spectroscopy*

Steady state fluorescence spectra were measured at (298.15 ± 0.5) K on an Agilent Cary Eclipse spectrofluorimeter. The fluorescence measurements employed 10 mm optical path length standard quartz cuvettes. For measurements of the intrinsic fluorescence spectral change of Yh due to the complexation with increasing HT-DNA concentration, the drug samples (dissolved in buffer) were excited at 250 nm. The excitation maxima of dug and the emission spectra scanned in the range of 300 to 450 nm. At least three consecutive measurements were taken for each sample and averaged out.

*Analysis of Binding Data*

Titration data were used to contrive Scatchard plots, *i.e.* by plotting *r*/*C*_f_ versus *r* for investigation; in which *r* is the number of drug molecules bound per mole of DNA and *C*_f_ is the free Yh concentration. In the case of normal titrations, the concentrations of free Yh (*C*_f_) and bound Yh (*C*_b_) are calculated using *C*_f_ = *C*(1 – ∝) and *C*b ) *C*_T_ – *C*_f_, respectively, where C_T_ is the total Yh concentration (15 *μ*M). The bound fraction of Yh (∝) was calculated using the equation, ∝ = (*A*_f_ – *A*)/(*A*_f_ – *A*_b_), where *A*_f_ and *A*_b_ are the absorbance of the free and fully bound Yh at the absorption maxima of Yh *i.e* at 352 nm, respectively, and *A* is the absorbance of Yh at 352 nm at any given point during the titration. As the binding for the interaction of Yh with different HT-DNA obtained here was linear, to calculate the binding parameters with higher accuracy, scatchard fitting models were used as described below.

Data were fitted using the following equation:

*r/C_f_ = K ×* (1– *n* × x) × ((1– *n×* x)/(1– (*n*–1) × x))^(*n*–1)                                                     (1)

where *r* is the number of alkaloid molecules bound per mole of DNA base pair and *C*_f_ is the molar concentration of the unbound alkaloids, *n* is the no of alkaloids bound per DNA base pair and *K* represents the affinity of the ligand’s for the binding site. Further the binding data were plotted as *r* vs *C*_f_ and analyzed by scatchard plot; Origin 8.5 was used for all the fitting analyses.

*Potassium iodide (KI) quenching experiments*

The quenching effect of KI was studied by adding stoichiometric small aliquots of potassium iodie (KI) stock solution to the three sets of the experiment. In one set of experiments, 50 μM of Yh was titrated with KI (0˗600 μM). In another set, the Yh-HT complex (1:1 ratio) was titrated with KI (0˗600 μM). The fluorescence intensity data were recorded, and then the quenching constants were calculated to form Stern-Volmer plots.

*Urea induced denaturation study*

This assay was done in three experimental setups. In the first experiment, 50 μM of Yh was titrated with urea (0-600 μM). In another one, the Yh to HT-DNA complex (1:1 ratio) was titrated with urea (0-600 μM) to record the emission spectra.

*Competitive drug displacement assay*

The competitive interaction between rhodamine B and Yh with HT-DNA was carried out as follows: fixed amounts of rhodamine B (10 μM) and HT-DNA (10 μM) were titrated by successive additions of Yh (2.5-12.5 μM). The emission was observed between 400-700 nm after exciting at 350 nm. The competitive interaction between ethidium bromide and Yh with HT-DNA was carried out as follows: fixed amounts of ethidium bromide (10 μM) and HT-DNA (10 μM) were titrated by successive additions of Yh (2.5-12.5 μM). The emission spectra were observed between 500-800 exciting at 475 nm.

*Effect of metal ions*

In this experiment, the complex of Yh (5μM) with metal ions (5μM) was titrated with varying concentrations of HT-DNA (0-15μM).The fluorescence spectra data were recorded in every case. The parameters were kept the same as in the steady-state fluorescence experiment.

*Molecular Modeling method*

AutoDock version 1.5.6 was used to do the molecular docking. Yh interaction with HT-DNA is investigated in this docking. According to the literature data, it could be assumed that the similar GC content of HT-DNA (PDB ID: 423D), and crystal structure were provided by the Protein Data Bank. GAUSSIAN 09 W program package was used to calculate the lowest energy-optimized structure of Yh (ligand) in the ground state which was obtained utilizing the hybrid method the B3LYP function and keeping the basis sets as 6-31G(d,p) (data not shown). The complete water molecule was deleted for both the DNA molecule construction, and polar hydrogen and Gasteiger charges were inserted into the macromolecule file. To contain the complete polymeric DNA molecule, the grid spacing was set at 0.375 Å with the dimension of 98 Å × 126 Å × 68 Å (X-Y-Z) for HT-DNA. The Lamarckian Genetic Algorithm was employed as a docking parameters algorithm in the docking operation, and some other parameters were set to the standard values provided by AutoDock 1.5.6. Based on the docked configuration with the lowest amount of energy, as calculated by the AutoDock scoring tool, the active binding was selected for each docking condition. PyMOL (The PyMOL Molecular Graphics System, Version 2.3.4, Schrödinger, LLC), UCSF Chimera 1.15 molecular graphics tool, and Discovery studio were used to visualize the docked position.

**Supplementary Figures:**

**
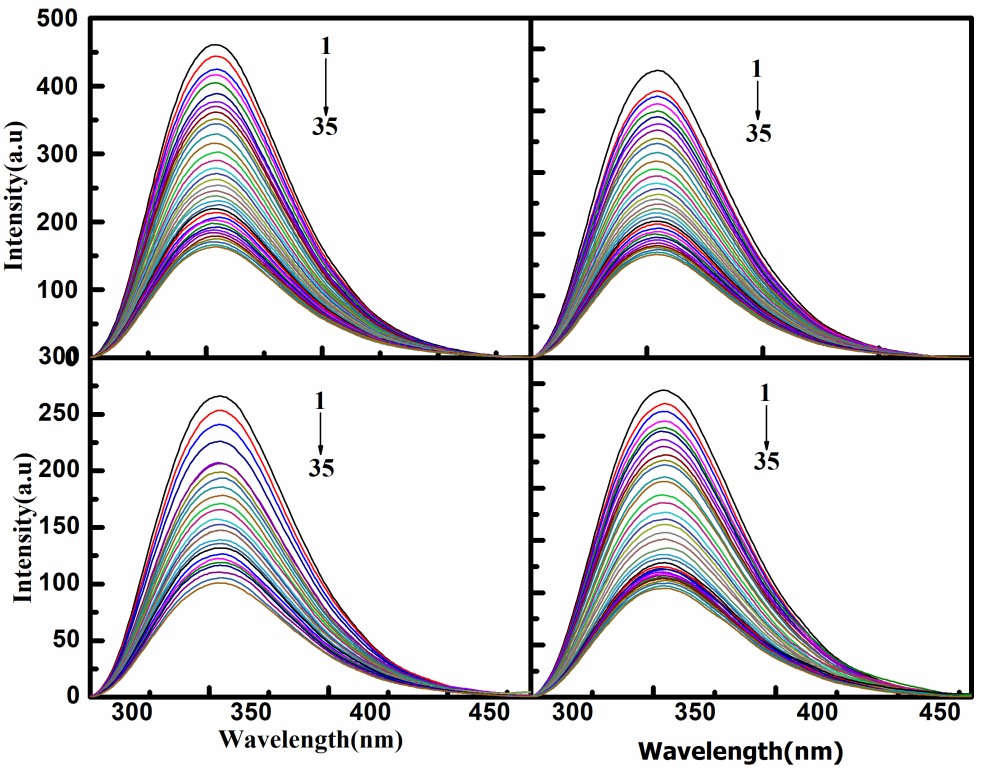
**

**(b)**

**(a)**

**(d)**

**(c)**

**Figure S1:** Fluorescence spectra of Yohimbine titration with CT DNA at different temperature: (a) 288.15 K (b) 293.15 K (c) 303.15 K and (d) 308.15 K. All the titrations are performed in presence of sodium cacodylate buffer pH 7.0.

**
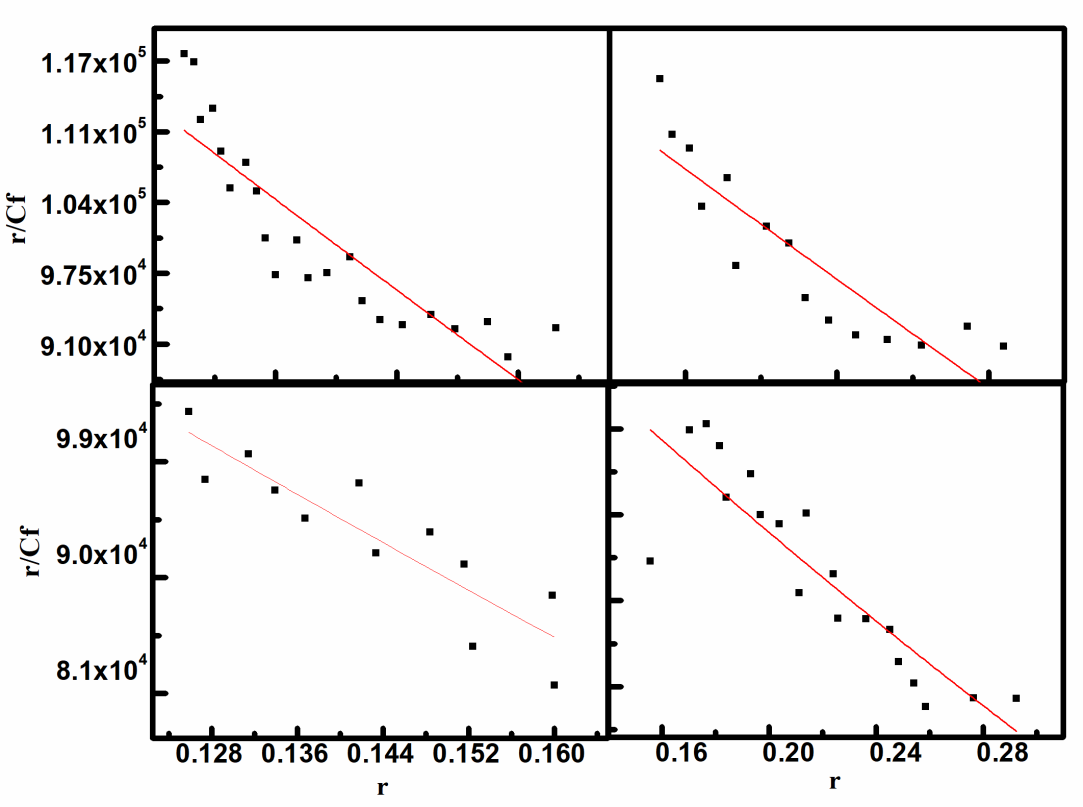
**

**(b)**

**(a)**

**(d)**

**(c)**

**Figure S2:** Scatchard Plot of Yohimbine titration with CT DNA at different temperature: (a) 288.15 K (b) 293.15 K (c) 303.15 K and (d) 308.15 K. All the titrations are performed in presence of sodium cacodylate buffer pH 7.0.

**
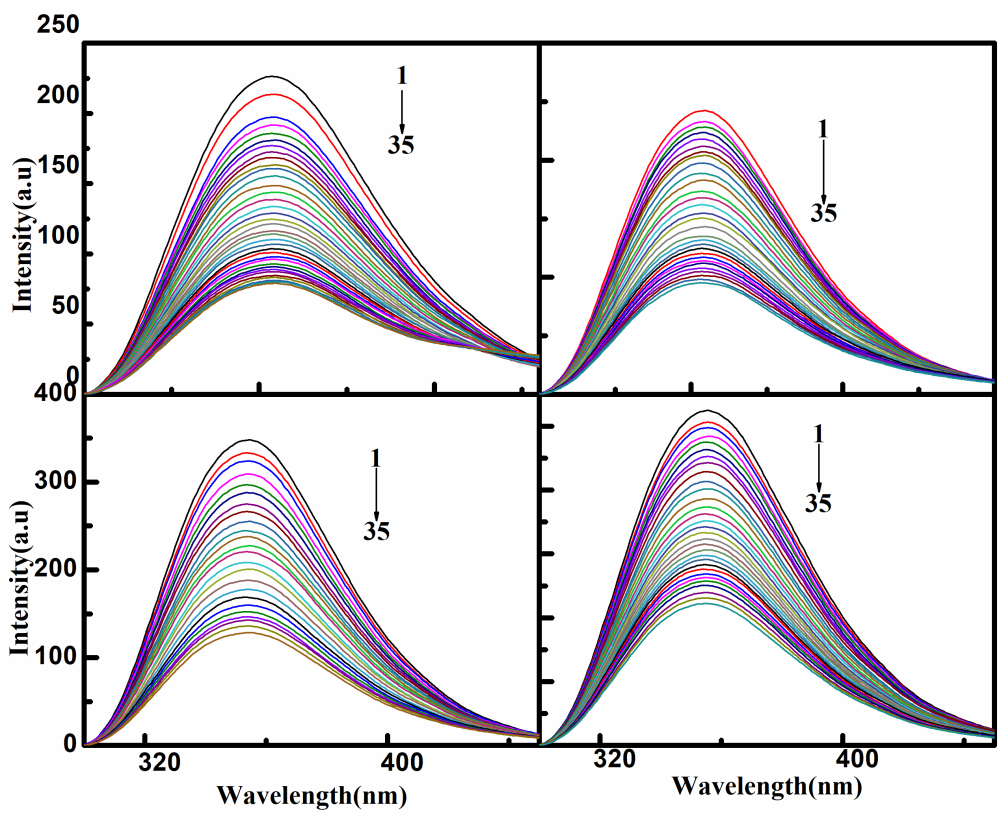
**

**(b)**

**(a)**

**(d)**

**(c)**

**Figure S3:** Fluorescence spectra of Yohimbine titration with CT DNA at salt concentration: (a) 20 mM (b) 30 mM (c) 50 mM and (d) 100 mM. All the titrations are performed at 298.15 K temperature.


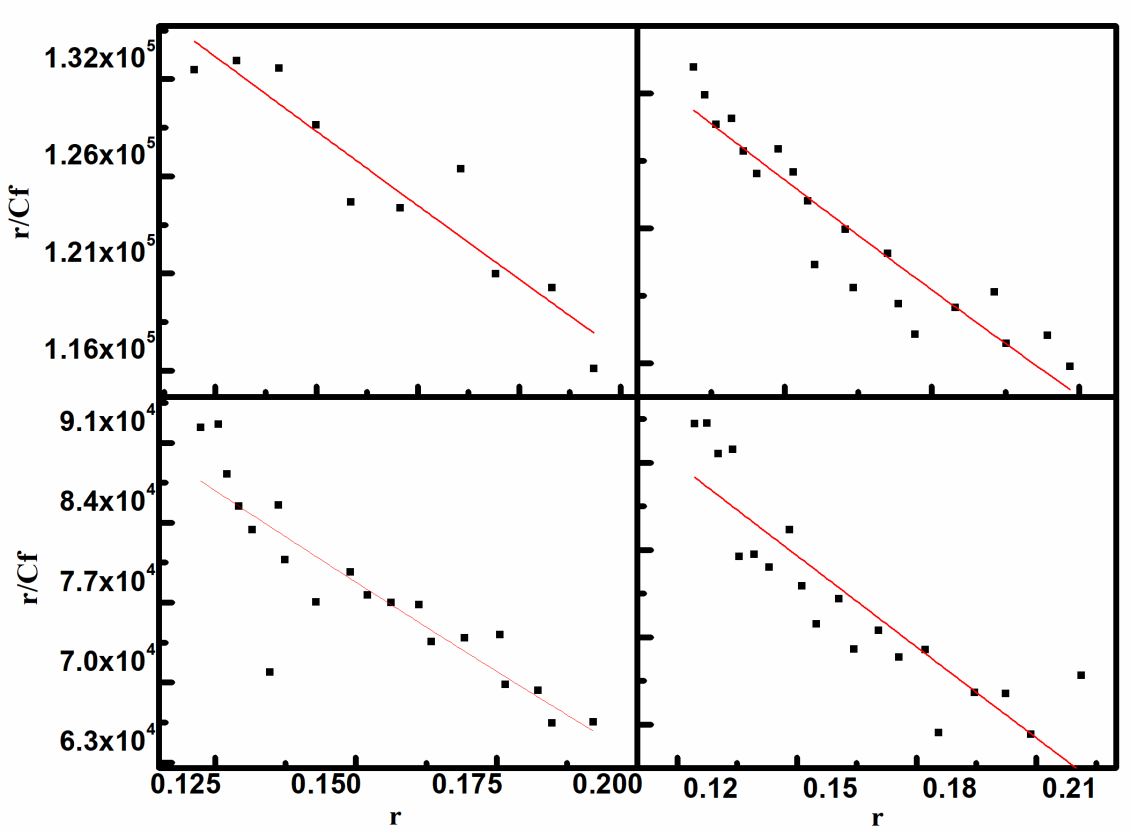


**(b)**

**(a)**

**(d)**

**(c)**

**Figure S4:** Scatchard Plot of Yohimbine titration with CT DNA at salt concentration: (a) 20 mM (b) 30 mM (c) 50 mM and (d) 100 mM. All the titrations are performed at 298.15 K temperature.


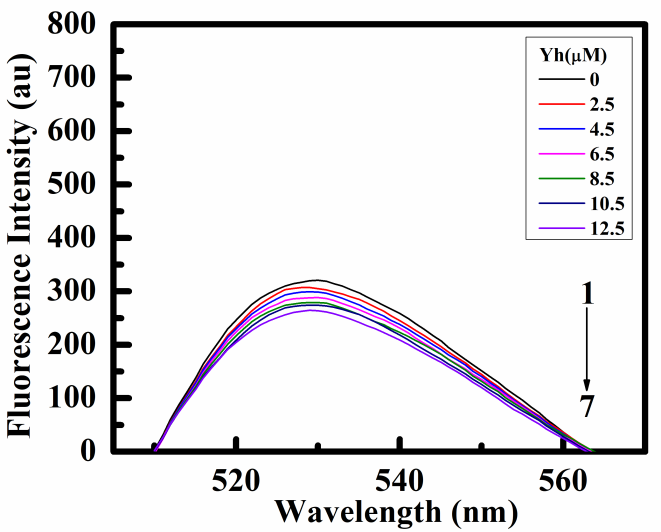


**Figure S5:** Fluorescence emission spectra obtained upon titration with Yh (0‒12.5 *μ*M) on the complex between HT-DNA and Acridine orange. Temperature = 298.15 K. All experiments were carried out in sodium cacodylate buffer (10 mM) of pH 7.0.
